# Cost-effectiveness of internet-supported cognitive behavioral therapy for university students with anxiety symptoms: A Markov-model analysis

**DOI:** 10.1371/journal.pone.0268061

**Published:** 2022-05-05

**Authors:** Joyce H. S. You, Scotty W. C. Luk, Dilys Y. W. Chow, Xinchan Jiang, Arthur D. P. Mak, Winnie W. S. Mak

**Affiliations:** 1 School of Pharmacy, Faculty of Medicine, The Chinese University of Hong Kong, Hong Kong SAR, China; 2 University Health Service, The Chinese University of Hong Kong, Hong Kong SAR, China; 3 Department of Psychiatry, Faculty of Medicine, The Chinese University of Hong Kong, Hong Kong SAR, China; 4 Department of Psychology, Faculty of Social Science, The Chinese University of Hong Kong, Hong Kong SAR, China; Yamaguchi University: Yamaguchi Daigaku, JAPAN

## Abstract

**Background and aim:**

High prevalence of anxiety symptoms has been reported globally in the university students. Cognitive behavioral therapy (CBT) is the recognized treatment for anxiety and is traditionally conducted face-to-face (f-CBT). The efficacy of internet-based CBT (i-CBT) for anxiety has been extensively studied, yet evidence on its cost-effectiveness is scarce. We aimed to evaluate the cost-effectiveness of guided low-intensity i-CBT for university students with mild anxiety symptoms from the societal perspective of Hong Kong.

**Methods:**

A 5-year Markov model was designed to compare outcomes of guided i-CBT and f-CBT in a hypothetical cohort of university students with mild anxiety symptoms. Model inputs of cost and healthcare resources associated with anxiety were retrospectively collected from a cohort of university students with anxiety symptoms. Clinical and utility model inputs were retrieved from published literature. Model outcome measures were anxiety-related total cost (including direct medical and indirect costs) and quality-adjusted life-year (QALY). Sensitivity analyses were performed to examine the robustness of base-case results.

**Results:**

In base-case analysis, i-CBT gained higher QALYs (2.9956 versus 2.9917) at lower total cost (US$6,101 versus US$6,246) than f-CBT. In one-way sensitivity analysis, the QALY gained by i-CBT was sensitive to the relative patient acceptance and adherence to CBT. In probabilistic sensitivity analysis, i-CBT was cost-effective in 90.9% of the time at the willingness-to-pay threshold of 138,210 per QALY (3× GDP per capita in Hong Kong). The probability of i-CBT to be cost-effective was 99.9% at a willingness-to-pay threshold of zero.

**Conclusions:**

Guided i-CBT appears to be cost-saving and effective for management of university students with mild symptoms of anxiety from the societal perspective of Hong Kong. The cost-effectiveness of i-CBT is highly subject to the individual acceptance and adherence of CBT delivered by the internet platform.

## Introduction

The mental health of university students is a global subject of concern. The World Health Organization (WHO) reported the findings of World Mental Health Surveys International College Student Project (n = 13,984) in 2018 that 31.4% of college students had 12-month mental disorders, and the 12-month prevalence of generalized anxiety disorder was 16.7% [1]. Poor mental health is associated with reduction in academic year percentage and increased dropout rate in higher education [2, 3]. Generalized anxiety increases the odds of severe role impairment (in home management, college-related and other work, close personal relationships, social life) by nearly 4-fold in university students [4]. The total direct medical expenditures on adults with anxiety disorders was approximated to be over USD33 billion in the US [5].

The World Mental Health Surveys showed only 24.6% of students would seek treatment, and the most commonly reported reason not to seek treatment was the preference of self-handling the problem alone (56.4%). Embarrassment was identified as an important barrier to treatment in students with generalized anxiety disorder [6]. A survey among 1,119 university students in Hong Kong found 54.4% of respondents to indicate symptoms of anxiety [7]. Another survey on 5,719 Chinese adults aged 16 to 75 years in Hong Kong also found low care-seeking rate (26.0%) among individuals with common mental disorders including anxiety and depression, especially in those aged 16 to 25 years [8].

Cognitive-behavioral therapy (CBT) is a problem-focused psychotherapy that integrates behavioral interventions such as behavioral activation, exposure therapy, and relaxation training into cognitive therapy [9]. CBT is the standard and effective management for symptoms of anxiety disorders [10], and has been traditionally administered in a face-to-face setting. Internet-based CBT interventions provide alternative options which are self-paced and minimize the embarrassment element in seeking-care. Growing evidence found positive effect of internet interventions for mental health in university students. A recent systematic review and meta-analysis (48 studies included) on internet interventions for mental health in university students reported that, in subgroup analysis, internet-based CBT had moderate effect on students with anxiety [11]. The guided internet-based CBT intervention was reported to improve the attributes of mental health among those who had completed the 8-week internet-based program among Hong Kong college students and young working adults [12].

Offering internet-based CBT could potentially improve the efficiency of health service delivery and maximize the favorable treatment outcomes from a health economic point of view. This study aimed to evaluate the potential cost-effectiveness of guided internet-based low-intensity CBT for university students with mild anxiety symptoms from the societal perspective of Hong Kong.

## Methods

### Model design

**Fig 1:**
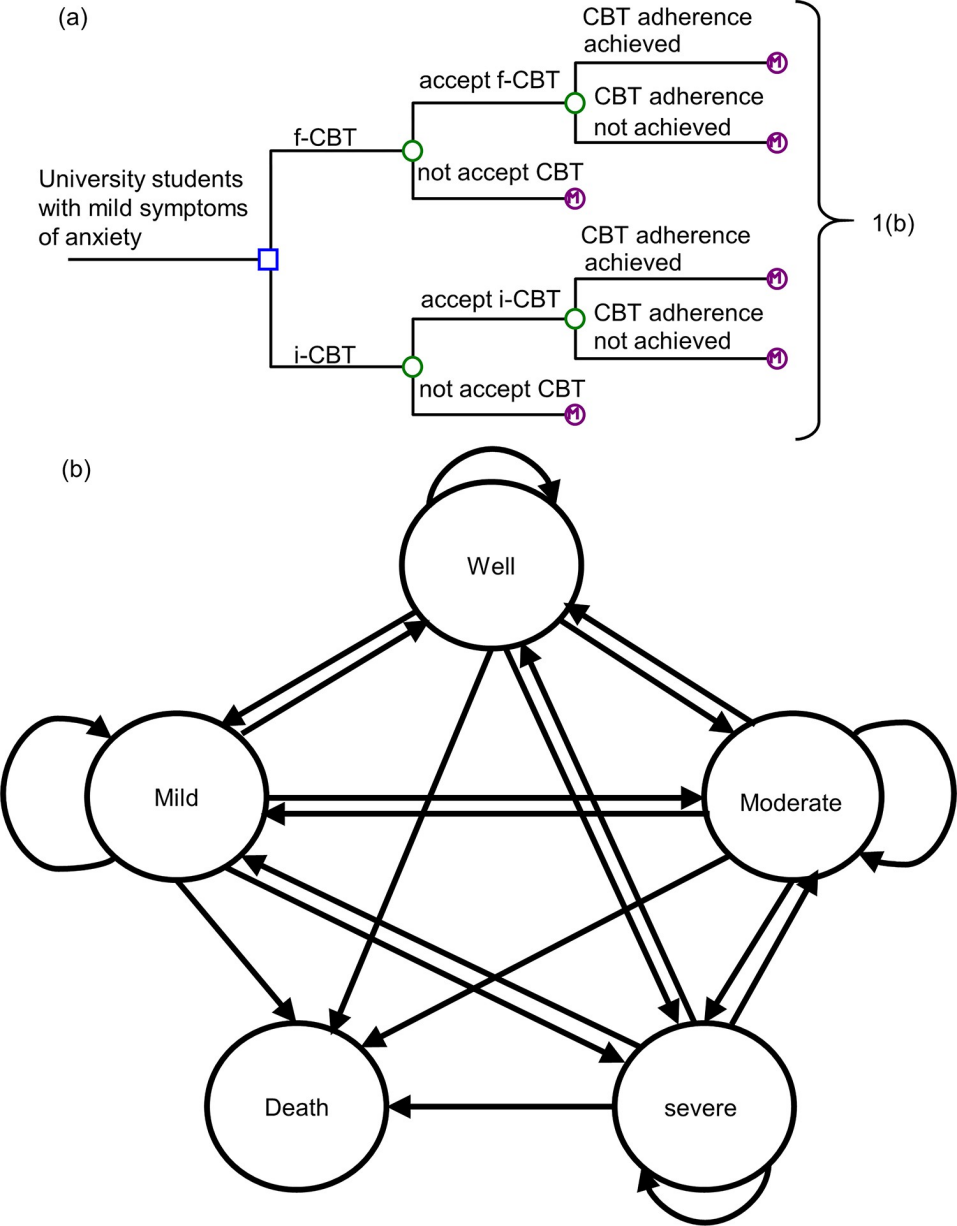
a, b. Simplified Markov model of university students with mild symptoms of anxiety. CBT: cognitive-behavioral therapy; f-CBT: face-to-face cognitive-behavioral therapy; i-CBT: internet-based cognitive-behavioral therapy.

A Markov model was developed to examine the outcomes of a hypothetical cohort of university students with mild symptoms of anxiety (**Fig 1**). Two delivery modes for low-intensity CBT were evaluated: (1) Guided internet-based CBT (i-CBT) and (2) face-to-face CBT (f-CBT) as comparator. In the Markov modeling, hypothetical subjects proceed through health states (Markov states) in the next model cycle according to transition probabilities. Five major Markov states were included: (1) Well, (2) mild symptoms, (3) moderate symptoms, (4) severe symptoms, and (5) death. The model outcome measures were anxiety-related total cost and quality-adjusted life-year (QALY). The model time frame was 5 years (with monthly cycle) to provide adequate analytic horizon for assessing the impact of the two delivery modes on long-term anxiety-related cost and QALYs in university students with mild symptoms of anxiety. It is recommended that the cycle length should be short enough to represent the frequency of clinical events and to reduce error in outcomes resulting from discretization, and therefore monthly cycle length was applied in the present model [13].

In this model, the low-intensity CBT program (up to 5–10 sessions, 30 minutes per session, over 9–12 weeks) [10] was delivered face-to-face or via internet platform. In the i-CBT group, the guided i-CBT was structured and scheduled to mirror face-to-face therapy. CBT was delivered by combining structured self-help intervention modules (presented via internet) and support of an identified therapist (who provided written feedback via email) [14]. In both study arms, the student might or might not accept the CBT. Those who accepted CBT might achieve adequate level of adherence with the CBT program. All students in the model might stay at the “mild” state, improve (to the “well” state), progress (to “moderate” or “severe” state), or die of all causes in each model cycle. The students who did not improve after the low-intensity CBT program were advanced to high-intensity face-to-face CBT (12–15 weekly sessions, 60 minutes per session), pharmacotherapy, or combination therapy [10].

### Clinical inputs

All model inputs are listed in **Table 1**. The clinical model inputs were retrieved from published literature. A MEDLINE search (year 2000–2021) was performed and found 240 articles using keywords such as “anxiety disorders”, “internet”, “online”, “cognitive-behavioral therapy”, “drug treatment”, “acceptance”, “adherence”, “remission”, and “progression”. Ten articles [15–24] were selected by selection criteria: (1) Reports written in English, (2) adults with symptoms of anxiety, (3) event rates of treatment effectiveness (remission and relapse) were reported, and (4) CBT acceptance or adherence rates were reported. Preferred studies were meta-analyses or randomized controlled trials. When multiple randomized trials were available for the same model input, the base-case value was estimated using the pooled average weighted against the number of patients in each study.

**Table 1:** Model inputs.

Parameters	Base-case value	Range of sensitivity analysis	Distribution	Reference
**Clinical Inputs**				
Acceptability of CBT				[15]
f-CBT	63.30%	50.6%-76.0%	Beta	
Relative difference of i-CBT *vs* f-CBT	1.19	0.952–1.428	Triangular	
Adherence to CBT				
f-CBT	77.5%	62.0%-93.0%	Beta	[16, 17]
Relative difference with i-CBT *vs* f-CBT	0.99	0.84–1	Triangular	[16–18]
Recovery				
No intervention	25.0%	20.0%-30.0%	Beta	[20, 21]
Low-intensity f-CBT	63.9%	51.1%-76.7%	Beta	[19]
Relative difference with i-CBT *vs* f-CBT	1.00	0.95–1.05	Triangular	[18]
Deterioration				
No intervention	17.4%	13.9%-20.9%	Beta	[20, 21]
Low-intensity f-CBT	5.3%	4.2%-6.4%	Beta	[19]
Relative difference with i-CBT *vs* f-CBT	1.00	0.95–1.05	Triangular	[18]
Monthly probabilities				
Well				
to mild	0.44%	0.35%-0.53%	Beta	[22]
to moderate	0.53%	0.42%-0.64%	Beta	[22]
to severe	1.66%	1.33%-1.99%	Beta	[22]
Mild				
to well	2.39%	1.91%-2.87%	Beta	[22]
to moderate	0.73%	0.58%-0.88%	Beta	[22]
to severe	0.67%	0.54%-0.80%	Beta	[22]
Moderate				
to well	4.48%	3.58%-5.38%	Beta	[22]
to mild	8.01%	6.41%-9.61%	Beta	[22]
to severe	2.75%	2.20%-3.30%	Beta	[22]
Severe				
to well	3.87%	3.10%-4.64%	Beta	[22]
to mild	3.14%	2.51%-3.77%	Beta	[22]
to moderate	3.91%	3.13%-4.69%	Beta	[22]
Hospitalization among severe anxiety cases	1.14%	0.91%-1.37%	Beta	[23]
Age-specific (18–22 years) all-cause mortality	0.0025%	0.0020%-0.0030%	Beta	[25, 26]
Relative risk of mortality with anxiety	1.66	1.56–1.77	Triangular	[24]
**Utility Inputs**				
Age in years	21	19–23	Triangular	Local
Age-specific utility	0.92	-		[28]
Remission utility	0.72	0.69–0.75	Triangular	[27]
Mild symptoms of anxiety	0.64	0.62–0.66	Triangular	[27]
Moderate symptoms of anxiety	0.60	0.58–0.62	Triangular	[27]
Severe symptoms of anxiety	0.53	0.50–0.56	Triangular	[27]
**Cost Inputs**				
Direct medical costs				
Hourly wage of clinical psychologists (US $)	52	42–62	Triangular	Local
Low-intensity CBT				[10]
Number of sessions	8	5–10	Triangular	
Duration of each session (minutes)	30	-		
High-intensity CBT				[10]
Number of sessions	14	12–15	Triangular	
Duration of each session (minutes)	60	-		
Platform overhead for i-CBT (US $)	19	15–23	Triangular	[29]
Percentage of time spent by psychotherapist in guided i-CBT (compared to f-CBT)	0.22	0.17–0.26	Triangular	[29]
Psychiatric outpatient (US $ per month)	220	185–348	Triangular	Local
Hospitalization (US $ per episode)	1278	1022–1534	Triangular	[30, 31]
Indirect cost				
Labor force participation rate (age 15–24 years)	36.4%	35.3–38.7	Beta	[32]
Unemployment rate	13.0%	11.4–14.4	Beta	[33]
Hourly wage of university student (US $)	10	8–12	Triangular	Local
Working hours per month	56	40–72	Triangular	Local

CBT: cognitive-behavioral therapy; f-CBT: face-to-face cognitive-behavioral therapy; i-CBT: internet-based cognitive-behavioral therapy.

The college students’ preference for internet-based (75.4%) versus in-person (63.3%) interventions for emotional needs were reported by a survey of 572 students [15]. The model adopted the reported preference for in-person interventions (63.3%) as the acceptance rate for f-CBT, and approximated the relative difference in acceptance of i-CBT versus f-CBT (75.4% versus 63.3%) to be 1.19-fold higher. The adherence rate of 77.5% to f-CBT was estimated from the results reported in two randomized controlled trials on (total 242) participants with symptoms of anxiety [16, 17]. A meta-analysis of 24 studies on i-CBT for adolescents and young adults found a mean adherence rate of 76.9% [18]. The relative difference in adherence to CBT delivered via internet versus in-person (76.9% versus 77.5%) were estimated to be 0.99 [16–18].

A local prospective outcome study reported the recovery (63.9%) and deterioration (5.3%) rates of low-intensity CBT for common mental disorders [19]. A systematic review on i-CBT demonstrated no significant difference in the effectiveness of CBT delivered via face-to-face and internet [18], and we therefore assumed a relative difference in the recovery and deterioration with i-CBT versus f-CBT to be 1.00. The recovery (25.0%) and deterioration (17.4%) rates in individuals who did not accept or adhere to the CBT were approximated from the outcome findings of the waiting list group reported by a health technology assessment study (including 7 systematic reviews of i-CBT for mild-to-moderate anxiety (n = 6) and depression (n = 1)) [20] and a review of internet-delivered psychological intervention [21], respectively. The probabilities of recovery and deterioration on initial low-intensity CBT (by f-CBT or i-CBT) were applied to the hypothetical students who were receiving the therapy. When the students (with mild anxiety at model entry) completed the f-CBT or i-CBT, the students might improve to “well”, deteriorate to “moderate” or “severe”, or stay in “mild”, according to the recovery and deterioration probabilities with low-intensity CBT.

The 3-month transition probabilities between states of well, mild symptoms, moderate symptoms, and severe symptoms following the completion of initial low-intensity CBT were obtained from the model inputs of a health economic analysis of CBT on generalized anxiety disorders [22], and were converted to monthly probabilities by the eigendecomposition approach [34]. The monthly probability of hospitalization in state of severe symptoms was approximated from the findings of a retrospective cohort study of health care utilization among patients with generalized anxiety disorders over a 6-month follow-up period [23]. The monthly all-cause mortality of individuals with anxiety symptoms was estimated from the age-specific all-cause mortality rate (0.0025%) in Hong Kong 2020 [25, 26] and the mortality rate ratio (1.66) associated with anxiety disorders [24].

### Utility and cost inputs

The QALY expected by a hypothetical student was estimated from the cumulative subject-time spent in each Markov health state and the corresponding utility reduction (comparing with the age-specific health utility). The age-adjusted utility of a Markov health state was approximated by: Age-specific health utility × health state-specific health utility. The health state-specific utility values were retrieved from a health-related quality of life study on patients with generalized anxiety disorders (N = 297) in the primary care setting [27]. The age-specific health utilities derived from the US national health measures and surveys were adopted [28]. The QALY was discounted to year 2022 by an annual rate of 3%.

The cost analysis was conducted from the societal perspective of Hong Kong with direct medical cost items (i-CBT, f-CBT, and psychiatric outpatient and inpatient care) and indirect cost items (productivity loss due to symptoms of anxiety). Direct cost of each resource item was estimated by (1) resource utilization and (2) unit cost of the item. All costs (in Hong Kong dollar) were presented in the US dollar using the currency conversion rate: US $1 = HK $7.8. The cost of f-CBT was approximated by the hourly wage of psychotherapists, the duration of each session, and the number of sessions per CBT course. The hourly wage of a registered psychotherapist was estimated from the salary scale at The Chinese University of Hong Kong (CUHK). The length and number of sessions were estimated based on the recommendation of low-intensity and high-intensity psychological interventions for generalized anxiety disorder in the NICE guideline [10]. The cost of i-CBT included the internet platform cost and staff cost. The overhead cost for the i-CBT platform was estimated to be US $19 [29]. The time spent by the psychotherapist per participant in the guided i-CBT was estimated to be 22% of the therapist’s time for f-CBT [29]. The cost per episode of inpatient psychiatric care was estimated using the daily cost of psychiatric hospitalization in Hong Kong [30] and the length of stay for anxiety disorders [31]. Health care resources utilization for outpatient psychiatric care were retrospectively estimated at University Health Service of CUHK. The CUHK is the only university in Hong Kong provides in-house clinical psychological counseling service and reimburses psychiatric consultations to students. Medical record review at the CUHK University Health Service was conducted for university students aged 18 years or above who received psychiatric consultation for symptoms of anxiety disorders (N = 50; 60% female; mean age 21 ± 2 years) during the period of 2017 to 2020. The study protocol was approved by The Joint CUHK-New Territories East Cluster Clinical Research Ethics Committee. The records of psychiatric service reimbursement were reviewed retrospectively to estimate the median cost per person per month (US $220). The costs accumulated over the 5-year model time frame were discounted with an annual rate of 3%.

Indirect cost analysis included productivity loss during the time spent in states of moderate and severe symptoms. Potential productivity loss was estimated using the friction cost approach by age-specific labor force participation rate and unemployment rate (reported by the Department of Census and Statistics of Hong Kong) [32, 33]. The percentage of employed students was estimated using the labor participation rate and unemployment rate in Hong Kong, and we assumed an employed student did not work when spending time (in the monthly cycle(s)) in the state of moderate to severe symptoms. The hourly wage and working hours per month adopted the student helper wage and allowable working hours for full-time students in the university, respectively. The friction period is not reported in Hong Kong, and was conservatively assumed as the duration of time with moderate-to-severe symptoms of anxiety disorders. All (direct and indirect) costs were discounted by an annual rate of 3% to year 2022.

### Cost-effective analysis and sensitivity analyses

All analyses were performed using TreeAge Pro 2021 (TreeAge Software Inc) and Excel 2016 (Microsoft Corporation). The base-case analysis compared the expected cost and QALY of i-CBT with the comparator (f-CBT). If a CBT strategy was more costly and gained higher QALYs than another alternative, incremental cost per QALY gained (ICER) for the more effective strategy was calculated by: Δcost/ΔQALYs. The WHO recommended that ICER less than 1× gross domestic product (GDP) per capita to be highly cost-effective and less than 3× GDP per capita to be cost-effective [35]. The GDP per capita of Hong Kong (US $46,070) in 2020 [36] was adopted as the willingness-to-pay (WTP) threshold in the base-case analysis. A treatment alternative was preferred if (1) it was effective in gaining QALYs at lower cost or (2) it was effective in gaining QALYs at higher cost and the ICER was less than the WTP threshold.

The range for sensitivity analysis was the 95% CI range or high/low values of the variable. If both the 95% CI range and high/low values were not reported, a range of ± 20% of the base-case value (with triangular distribution) was applied as the range for sensitivity analysis. One-way sensitivity analysis on all model inputs was performed to identify influential factors with threshold value. Probabilistic sensitivity analysis was performed by Monte Carlo simulations. Cost and QALY of each study arm were recalculated 10,000 times by randomly drawing each model input from the parameter-specific distribution (**Table 1**). The incremental cost and incremental QALYs of i-CBT versus f-CBT were presented in a scatter plot. The probability of each strategy to be accepted as cost-effective was determined over a range of WTP from zero to 3× GDP per capita of Hong Kong in the acceptability curves.

## Results

### Base-case analysis

The expected base-case deterministic (per person) direct, indirect, and total costs, and QALYs of i-CBT and f-CBT are shown in **Table 2**. The i-CBT gained higher QALYs than f-CBT by 0.0039. The direct and indirect costs of i-CBT were both lower than those of f-CBT (and i-CBT saved total cost by US $145). Comparing with f-CBT, the i-CBT group gained higher QALYs at lower cost, and i-CBT was therefore the preferred strategy in the base-case analysis.

**Table 2:** Base-case (deterministic) and probabilistic results.

Strategy	Direct cost (US $)	Indirect cost (US $)	Total cost (US $)	QALY gained	Increment cost (US $)	Incremental QALY
**Base-case (deterministic)**						
**i-CBT**	4,317	1,784	6,101	2.9956	- 145	0.0039
**f-CBT**	4,454	1,792	6,246	2.9917	-	-
**Probabilistic**						
**i-CBT (95%CI)**	4,532 (4,525–4,539)	1,791 (1,786–1,796)	6,322 (6,313–6,330)	2.9941 (2.9934–2.9947)	-119 (-118 to -120)	0.00267 (0.00262–0.00271
**f-CBT (95%CI)**	4,646 (4,639–4,653)	1,796 (1,791–1,802)	6,441 (6,432–6,450	2.9914 (2.9908–2.9920)	-	-

Base-case (deterministic) and probabilistic results are presented as costs and QALY gained per person

f-CBT: face-to-face cognitive-behavioral therapy; i-CBT: internet-based cognitive-behavioral therapy; QALY: quality-adjusted life-year

### Sensitivity analyses

**Fig 2:**
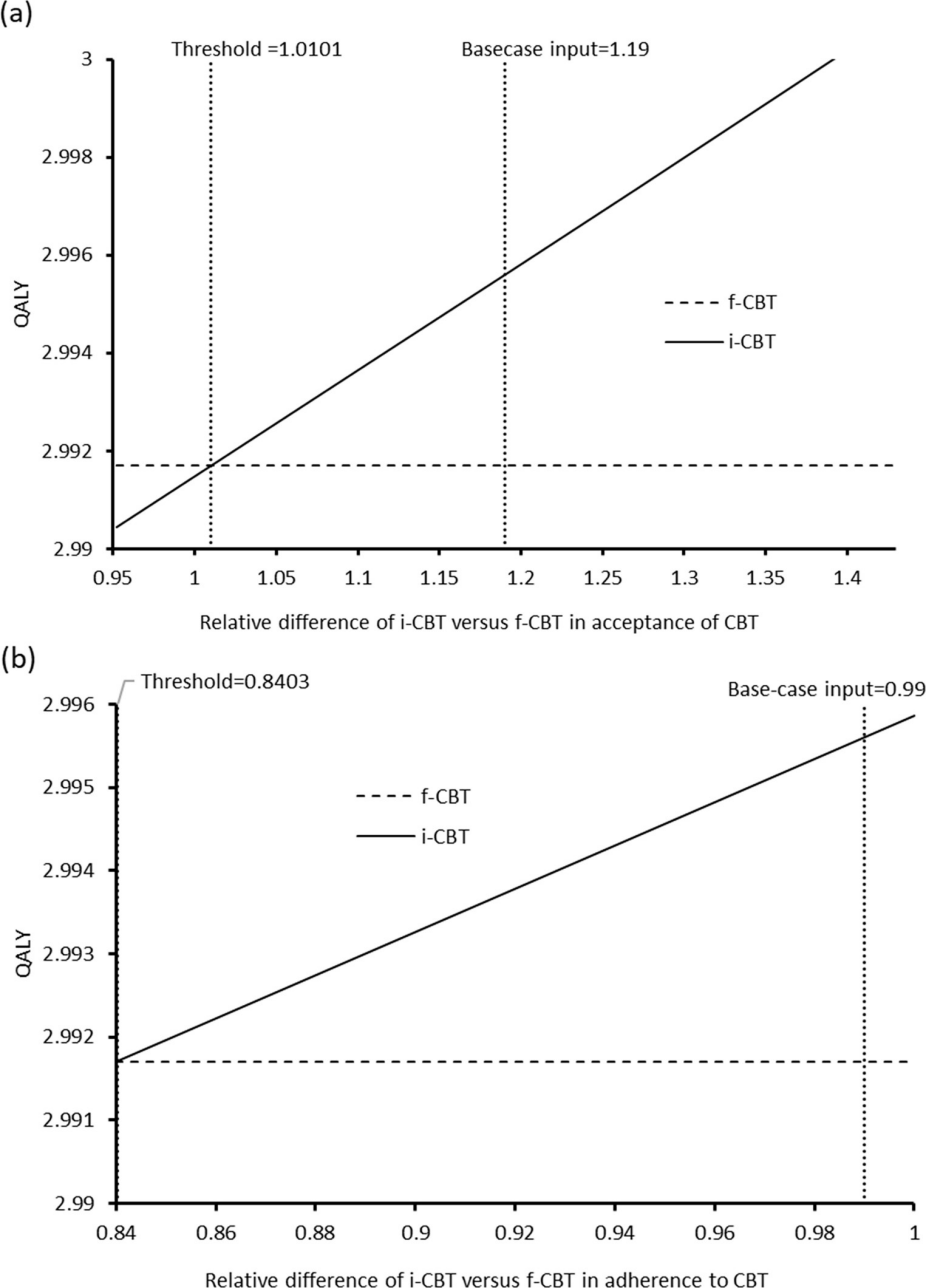
One-way sensitivity analysis on change of QALY against relative difference of i-CBT versus f-CBT in (a) acceptance of CBT and (b) adherence to CBT. CBT: cognitive-behavioral therapy f-CBT: face-to-face cognitive-behavioral therapy; i-CBT: internet-based cognitive-behavioral therapy; QALY: quality-adjusted life-year.

The one-way sensitivity analysis showed that the i-CBT group remained to be less costly than the f-CBT group throughout the variation of all model inputs, and no model input with threshold value was identified. The base-case results of QALYs were found to be sensitive to two model inputs with threshold value: Relative difference of i-CBT versus f-CBT in (1) acceptance of CBT and (2) adherence to CBT (**Fig 2**). When the base-case inputs reduced to the threshold value (relative difference of i-CBT versus f-CBT in acceptance of CBT <1.0101 (base-case value: 1.19); or adherence to CBT <0.8403 (base-case value: 0.99)), the i-CBT gained lower QALYs than the f-CBT.

**Fig 3:**
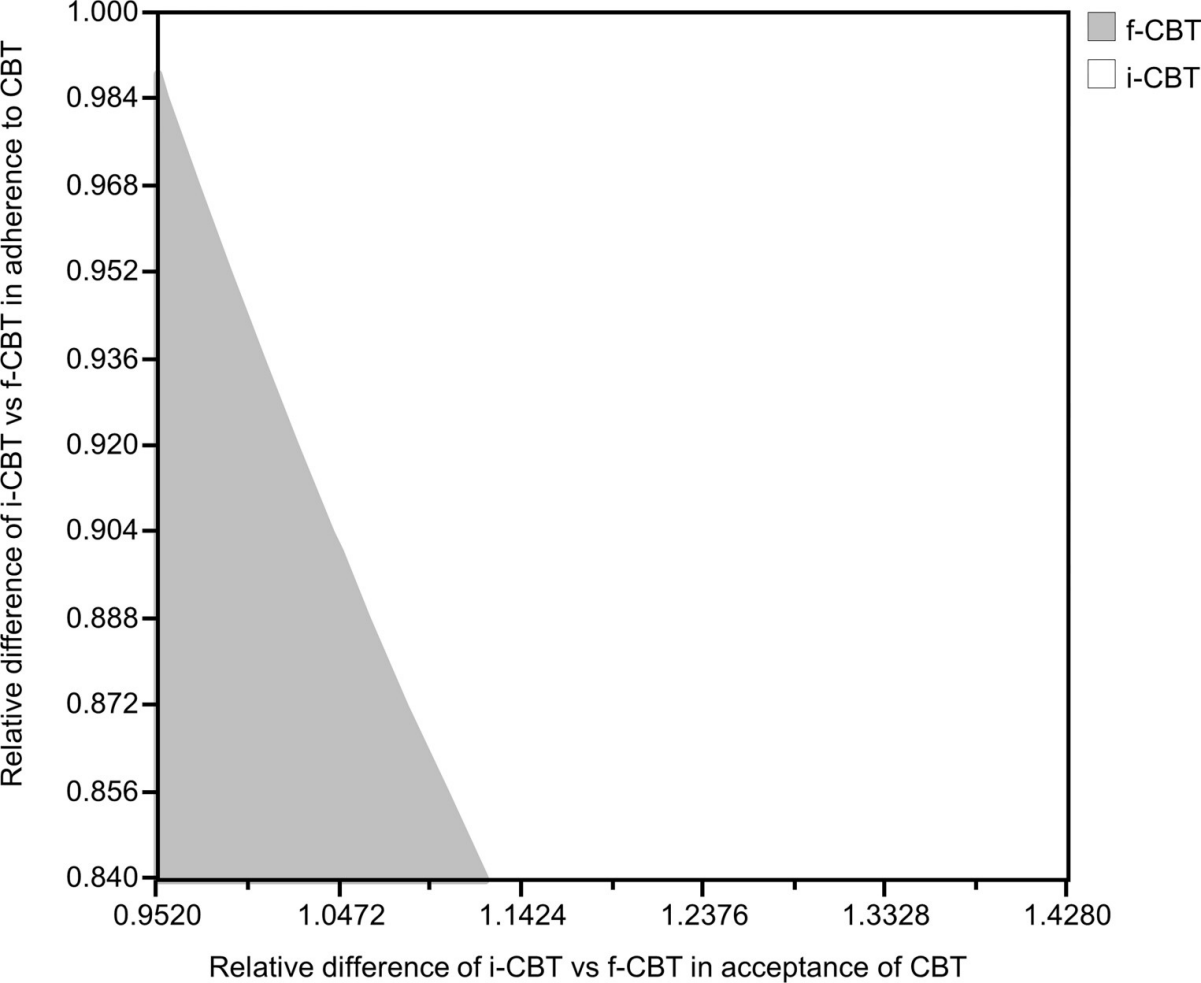
Two-way sensitivity analysis of acceptance and adherence to CBT on cost-effectiveness of i-CBT. Threshold line divided the grey zone and white zone; combinations of variables on threshold line had the same cost-effectiveness for i-CBT and f-CBT; white zone: combinations of variables leaded to i-CBT to be cost-effective; grey zone: combinations of variables leaded to f-CBT to be cost-effective. CBT: cognitive-behavioral therapy; f-CBT: face-to-face cognitive-behavioral therapy; i-CBT: internet-based cognitive-behavioral therapy; QALY: quality-adjusted life-year.

A two-way sensitivity analysis was further conducted to examine the interacting effect of these two influential model inputs on the cost-effectiveness of i-CBT. In **Fig 3**, the area in white indicated the combinations of model input values for i-CBT to be cost-effective at the willingness-to-pay threshold of US $46,070 per QALY. The grey area indicated the combinations of model input values to find f-CBT to be the cost-effective option. At low relative difference (0.840) in adherence to CBT (i-CBT versus f-CBT), the i-CBT needed to achieve relative difference in acceptance of CBT >1.13 to be cost-effective. If the relative difference in acceptance to CBT was 0.952 with i-CBT (versus f-CBT), the relative difference in adherence of CBT needed to be >0.99 for i-CBT to remain cost-effective.

**Fig 4:**
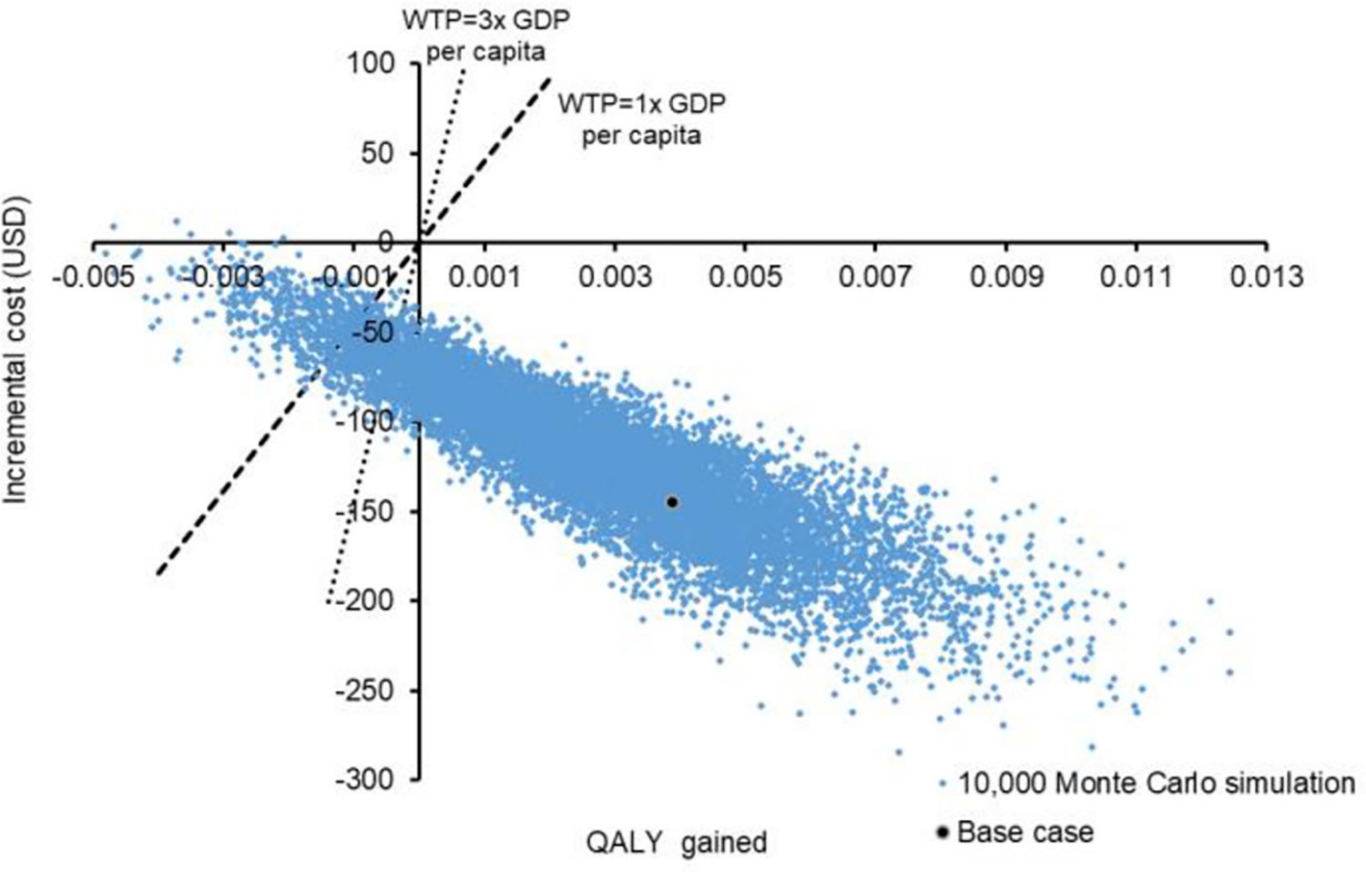
Scatterplot of incremental cost against QALYs gained by i-CBT versus f-CBT. f-CBT: face-to-face cognitive-behavioral therapy; i-CBT: internet-based cognitive-behavioral therapy; QALY: quality-adjusted life-year.

**Fig 5:**
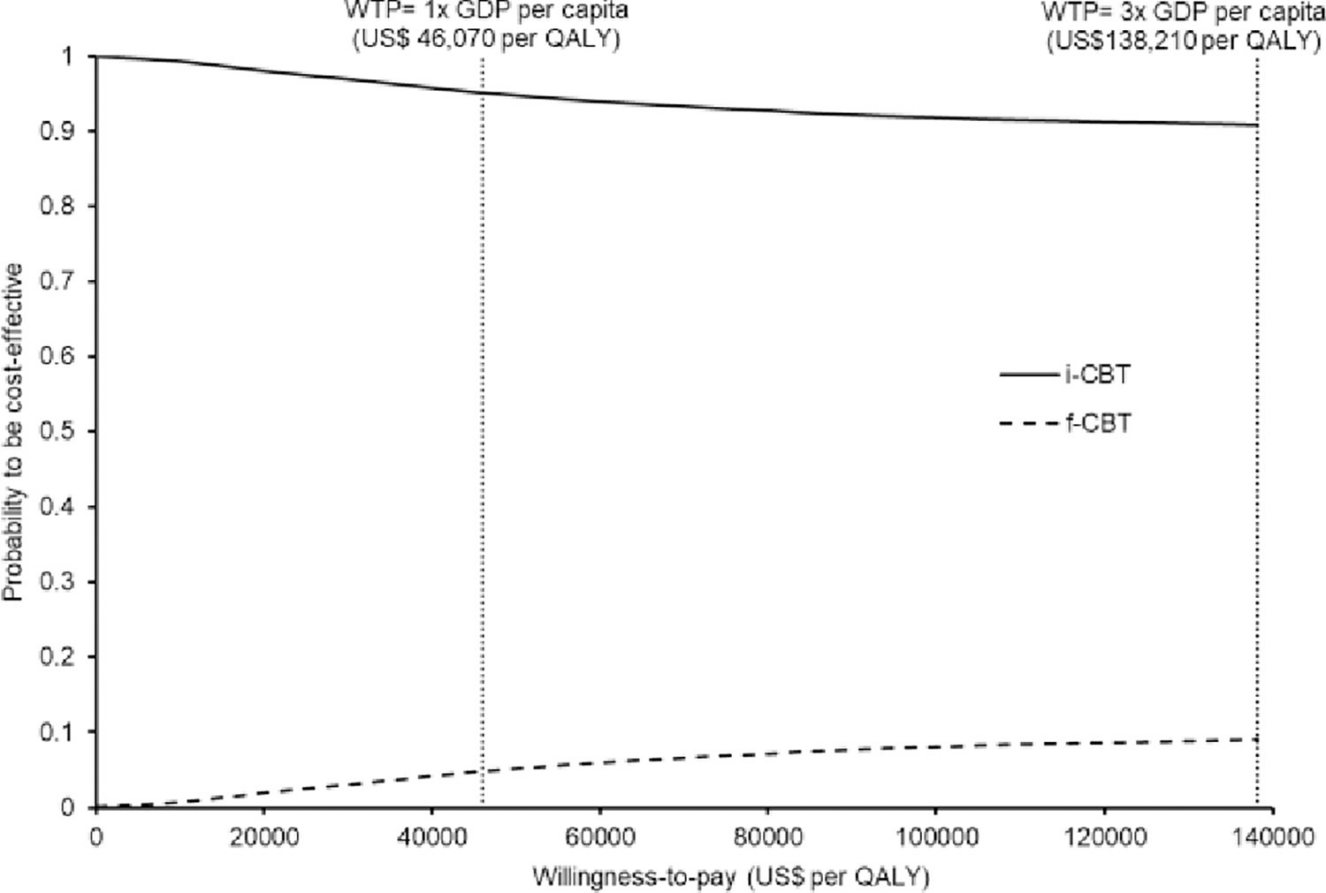
Acceptability curves of i-CBT and f-CBT to be cost-effective against willingness-to-pay. f-CBT: face-to-face cognitive-behavioral therapy; i-CBT: internet-based cognitive-behavioral therapy.

Probabilistic sensitivity analysis was performed by recalculating the total cost and QALYs 10,000 times using Monte Carlo simulations. The probabilistic (per person) direct, indirect, and total costs, and QALYs of i-CBT and f-CBT are shown in **Table 2**. The incremental cost against incremental QALYs gained by the i-CBT versus f-CBT were shown in a scatter plot (**Fig 4**). When compared with the f-CBT, the i-CBT gained higher QALY by 0.00267 (95% CI 0.00262 to 0.00271, p<0.001) with a cost-saving of US $119 (95% CI US $118 to US $120, p<0.001). The probabilities of each strategy to be accepted as cost-effective were presented in the acceptability curves (**Fig 5**). The i-CBT was cost-effective in 90.9% of the time at the willingness-to-pay threshold of 138,210 per QALY (3× GDP per capita in Hong Kong). The probability of i-CBT to be cost-effective was 99.9% at a willingness-to-pay threshold of zero.

## Discussion

This is the first cost-effectiveness analysis to evaluate the impact of guided i-CBT on the outcomes of university students with mild anxiety symptoms, measured as total cost (including direct medical and indirect costs) and QALYs, from the societal perspective of a high-income city. Compared to the f-CBT, offering guided i-CBT to manage students with mild anxiety symptoms reduced cost (by US $145 per case) and gained additional QALYs (by 0.0039 per case) in a five-year time horizon. The QALY gain was generated by improved acceptance rate of CBT associated with i-CBT versus f-CBT and the consequently enhanced recovery. The cost-saving in the i-CBT arm was primarily due to lowered requirement of clinical psychologist staff-time and reduction in deteriorated cases (resulting in reduced utilization of outpatient and inpatient psychiatric care). The one-way sensitivity analysis found the cost-saving of i-CBT to be highly robust and no influential parameter (with threshold value) was identified throughout variation of all model inputs. The additional QALY gained by i-CBT was sensitive to the relative acceptance and adherence to CBT with internet-based delivery versus face-to-face model of care. It is well reported that i-CBT received higher acceptance by individuals, yet the adherence to CBT is weakened on the internet platform [15, 18]. The two-way sensitivity analysis mapped out the combinations of these two influential factors for i-CBT to be accepted as the cost-effective option. The results of probabilistic sensitivity analysis further supported the guided i-CBT to be highly likely (>90%) accepted as the cost-effective option over a wide range of WTP threshold in the 10,000 Monte Carlo simulations.

Despite the well-reported effectiveness of i-CBT for anxiety in the clinical setting, the health economic data were scarce. Kumar et al. examined the cost-effectiveness of a mobile application-delivered CBT for adults with generalized anxiety disorder in the US [22]. The results of the US analysis were similar to the present study that the mobile CBT was cost-saving and gained higher QALYs when compared with traditional CBT. The acceptance and adherence of CBT were not considered in the US study. The findings of the present analysis therefore supported the previously reported cost-effectiveness of internet-based CBT in the US, and also added critical information on the key influential factors (relative acceptance and adherence to CBT with internet-based delivery).

The mental health of university students has become a growing global concern amid the pandemic. With the outbreak of the COVID-19 pandemic and the periodic lockdown measures to contain the spread of disease, psychological reactions to the pandemic have become prominent since. University campuses have been closed down for prolonged period of time during 2020 and have only reinstated few in-person learning activities in early 2021. Surveys of university students (in Spain, China, and Hong Kong) found substantial respondents to report anxiety (45%) [37–39]. The anxiety symptoms were also reported in the Hong Kong secondary school students [40]. Most individuals who experienced emotional distress during disasters eventually regain usual functioning level, yet some of them develop chronic mental disorders [41]. The unattended or unresolved mental health symptoms will possibly sustain a long-term impact when the affected secondary school students advance to university study. It is therefore anticipated that the impact of COVID-19 on mental health of university students will linger on into the postpandemic period. The demand for CBT in the university campus is raising since the reopening of face-to-face learning, resulting in the longer waiting time to face-to-face counseling. The COVID-19 pandemic has imposed major burden as well as barrier on the operation of health care systems around the world. It disrupts the provision of in-person mental and medical care, and also causes patients to delay and avoid seeking care [42]. COVID-19 has been reported to be a factor associated with avoiding medical consultation in Hong Kong [43].

A recent systematic review found a mental health awareness rate of 73% among Chinese university students [44], lower than the goal (80% awareness among students) set by the China national mental health work plan [45]. The suboptimal mental health awareness is associated with low care-seeking rate [44]. The demand for internet-based CBT has been increasing during the pandemic, indicated by a markedly increase in registration of online CBT and the correspondingly improved health anxiety [46]. Online guided and un-guided interventions for university students are increasingly examined and found to be a feasible and efficacious [47, 48]. The telemental health bridges the physical distance between individuals with mental health needs and service providers. This health economic analysis provided essential evidence to assist policy makers on making informed decision of resource allocation for evaluating the uptake and effectiveness of telemental health services. The nature of present model-based analysis is exploratory, and the findings are highly indicative for supporting further research on the demand, feasibility, acceptance, adherence, and effectiveness of i-CBT for university students with mild symptoms of anxiety in Hong Kong.

There were some limitations in the present study. Model-based analyses are in general subject to uncertainty of model inputs. Most of the clinical model inputs were extracted from international clinical trials, and also few of which were retrieved from trials of general individuals with anxiety symptoms and individuals with social phobia. Also, the efficacy generated by clinical trials is usually higher than real-world effectiveness. The sources of the clinical inputs might affect the generalizability of model results to university students in Hong Kong. Rigorous sensitivity analyses were therefore performed to examine the impact of model input uncertainty on the base-case results. The consensus of WTP threshold has not yet reached in Hong Kong, and a GDP-based WTP threshold was adopted in the present study to evaluate the cost-effectiveness of the intervention. The use of GDP-based WTP threshold provides information to guide policy-makers on value for money and reflect the monetary value of the benefits, yet a fixed WTP threshold should not be the sole factor to decide the acceptance of a new intervention [49]. Affordability, budget impact and implementation feasibility should also be considered together with cost-effectiveness findings in the decision-making process [50]. An intervention with high ICER was reported to be associated with decreased odds of receiving cost-effectiveness recommendation [51]. We therefore evaluated the probabilities to be accepted as cost-effective against a wide variation of WTP threshold (zero to 3x GDP per capita) and found the cost-effectiveness of i-CBT remained robust. The present study used a simplified Markov model to represent CBT acceptance and adherence, and the corresponding outcomes in students with deteriorated anxiety symptoms. The negative impact of waiting time for face-to-face CBT was not incorporated in the present model, and might therefore underestimate the cost-effectiveness benefits of internet-based CBT. Our search of model inputs was performed in English publications, and possibly missed the relevant data reported in non-English languages.

## Conclusions

In conclusion, implementing guided i-CBT to the student health management appears to be cost-saving and effective for university students with mild symptoms of anxiety from the societal perspective of Hong Kong. The cost-effectiveness of i-CBT is highly subject to the individual acceptance and adherence of CBT delivered by the internet platform.
